# Determinants of organ donation knowledge and attitudes among university students in Oman: a secondary analysis using logistic regression and machine learning

**DOI:** 10.3389/fpubh.2026.1841389

**Published:** 2026-05-28

**Authors:** Asli Altuntas, Sami Akbulut, Zeynep Kucukakcali

**Affiliations:** 1 Transplantation Coordinator Program, Liver Transplant Institute, Inonu University, Malatya, Türkiye; 2 Department of Surgery and Liver Transplantation, Faculty of Medicine, Inonu University, Malatya, Türkiye; 3 Department of Biostatistics and Medical Informatics, Faculty of Medicine, Inonu University, Malatya, Türkiye

**Keywords:** attitude, knowledge, logistic regression, machine learning, organ donation, university students

## Abstract

**Background:**

Organ donation remains insufficient globally, particularly in settings where sociocultural and religious perceptions shape decisions. This study examined knowledge and attitudes toward organ donation among university students and identified determinants using multivariable logistic regression and machine learning (ML) models.

**Methods:**

This secondary cross-sectional study included 2,125 students. Knowledge and attitude scores were dichotomized at a 60% threshold. Variables with *p* < 0.05 in chi-square analyses were considered for multivariable logistic regression. Three ensemble ML models were trained using stratified 80/20 datasets and evaluated using accuracy, sensitivity, specificity, precision, recall, F1-score, and ROC-AUC. Feature-importance analysis assessed model interpretability.

**Results:**

Although awareness of organ donation was high (98.5%), only 17.6% demonstrated good knowledge and 28.6% exhibited a high attitude level. Multivariable logistic regression showed that a master’s degree was associated with lower odds of good knowledge (OR = 0.39, *p* = 0.012), whereas knowing someone requiring transplantation (OR = 1.64, *p* = 0.020), believing that Islamic teachings permit donation (OR = 1.29, *p* = 0.033), and reporting other reasons for refusing organ donation (OR = 1.53, *p* = 0.023) increased knowledge levels. AdaBoost achieved the highest overall accuracy for knowledge classification (0.82), although this result should be interpreted cautiously because of its limited ability to identify participants with good knowledge. Feature-importance analysis identified personal exposure to transplantation, educational background, and refusal-related considerations as key predictors. Higher attitude scores were associated with perceiving organ donation as life-saving and religiously compatible (OR = 2.60, *p* < 0.001), altruistic motivation (OR = 1.60, p < 0.001), media influence (OR = 1.91, *p* < 0.001), belief that Islamic teachings permit organ donation (OR = 1.61, *p* < 0.001), financial motivation (OR = 2.70, p < 0.001), and reporting other reasons for refusing organ donation (OR = 1.66, *p* = 0.011), while lack of awareness was the strongest negative predictor (OR = 0.23, *p* < 0.001) along with perceived irrelevance, fear, perceived religious prohibition, and restrictive donation preferences. For attitude classification, AdaBoost also achieved the highest accuracy (0.79), while feature-importance analysis identified lack of awareness, perceived irrelevance, media influence, and life-saving/religious alignment as the most influential predictors.

**Conclusion:**

Despite high awareness, notable gaps remain between knowledge, attitude, and behavioral intention. Logistic regression and ML modeling showed partially overlapping patterns, with both approaches identifying shared determinants of knowledge and attitudes toward organ donation. These findings suggest ML may complement conventional regression by supporting the predictive relevance and interpretability of key behavioral and perceptual factors.

## 1 Introduction

Organ transplantation is widely recognized as one of the most effective life-saving therapeutic options for patients with end-stage organ failure, owing to major advances in surgical techniques, perioperative care, and immunosuppressive therapies (1, 2). Despite these clinical achievements, the global shortage of transplantable organs remains a critical public health challenge, with substantial medical, ethical, and societal implications (3, 4). Organ donation rates vary considerably across countries and sociocultural contexts, where religious beliefs, ethical perceptions, and cultural norms strongly influence individuals’ willingness to donate organs and their donor registration behaviors (5, 6). As the demand for transplantation continues to increase worldwide, the persistent imbalance between organ supply and demand results in prolonged waiting times and substantially increased mortality rates among patients awaiting transplantation (7–9).

A growing body of evidence indicates that insufficient knowledge, misconceptions, and uncertainty regarding religious permissibility are among the most frequently reported barriers to organ donation, particularly in Muslim-majority societies (10–13). Even in settings where religious authorities formally endorse organ donation, inadequate public awareness of these religious rulings may lead to hesitation and unfavorable attitudes toward donation. Therefore, improving organ donation rates requires not only strengthening organ transplant infrastructure but also developing culturally sensitive educational strategies that address knowledge gaps, clarify religious perspectives, and promote positive social attitudes toward organ donation (14–17).

University students represent a strategically important population for organ donation awareness initiatives. As relatively well-educated, socially active, and digitally connected individuals, university students may act as influential information transmitters within their families and communities and thereby shape broader societal perceptions (5, 18). Nevertheless, several studies conducted in different regions have reported that university students frequently possess fragmented or inadequate knowledge about organ donation and transplantation, which may negatively influence donation intentions, willingness to register, and advocacy behaviors (19–21).

In Muslim communities, religious interpretation plays a particularly influential role in shaping perceptions of organ donation (13, 22, 23). Although numerous Islamic scholars and international religious councils have issued fatwas supporting the permissibility of organ donation under specific ethical conditions, misinterpretations of religious teachings and uncertainty regarding religious approval continue to persist among segments of the public (24, 25). For example, a study conducted among medical students in Egypt reported that approximately one-fifth of negative attitudes toward organ donation were related to perceived religious concerns, underscoring the importance of integrating religious clarification into educational interventions (26).

Despite the growing literature on public perceptions of organ donation, analytical studies specifically examining university students in Muslim-majority countries remain relatively limited, particularly those employing advanced analytical approaches to explore determinants of knowledge and attitudes (27, 28). Considering that university students may serve as future healthcare professionals, educators, and policy influencers, understanding the factors that shape their knowledge and attitudes toward organ donation is essential for designing effective awareness strategies and educational interventions.

In recent years, artificial intelligence (AI) and its subfield, machine learning (ML), have increasingly been applied in transplantation and organ donation research. Several studies have utilized ML models to predict donor registration behavior, family consent to organ donation, and willingness to donate by identifying complex interactions among demographic, psychosocial, and cultural variables. Such ML-based approaches enable the identification of non-linear relationships and hidden interaction patterns that may not be fully captured through conventional statistical analyses alone, thereby offering complementary insights into behavioral determinants of organ donation (29–32). Nevertheless, although ML models have been applied to several organ donation–related behavioral outcomes, studies that integrate multivariable regression analysis with ensemble ML models to jointly evaluate knowledge and attitudes toward organ donation among university students in Muslim-majority settings remain limited.

Accordingly, the present study conducted a secondary analysis of an open-access dataset derived from university students in Oman, a Muslim-majority country, to comprehensively evaluate knowledge and attitudes toward organ donation. By integrating multivariable logistic regression with ensemble ML models, the study sought to identify sociodemographic and perceptual determinants associated with knowledge and attitude levels. This combined approach was intended to provide complementary explanatory and predictive insights that may inform targeted educational strategies for improving organ donation awareness and engagement.

## 2 Materials and methods

### 2.1 Study design and data source

This study was conducted as a secondary analysis of an openly accessible dataset derived from a previously published cross-sectional observational survey investigating knowledge and attitudes toward organ donation and transplantation among university students in Oman (5). The original survey was carried out between August 2021 and February 2022 at Sultan Qaboos University and included students from nine academic faculties: Medicine and Health Sciences, Engineering, Agricultural and Marine Sciences, Economics and Political Science, Science, Nursing, Law, Education, and Arts and Social Sciences.

In the original study, eligible participants included all local students of any age and academic year who were actively enrolled during the study period. Exclusion criteria comprised non-local students and those enrolled in the first semester of the foundation program. Of the 2,173 students who initially participated, 48 were excluded based on predefined eligibility criteria, resulting in a final analytic sample of 2,125 students.

Due to COVID-19 pandemic restrictions, data were collected using an online questionnaire administered through Google Forms. The survey link was distributed via institutional email lists and social media platforms, and participation was voluntary and self-administered. The questionnaire included both self-developed items and items adapted from previously published literature. It was available in both English and Arabic to ensure accessibility.

The dataset was obtained from the supplementary materials accompanying the original open-access publication and consisted of anonymized individual-level responses. The original article was published under the Creative Commons Attribution License, which permits use, distribution, and reproduction provided that the original work is properly cited. Because the dataset was publicly accessible and contained no personally identifiable information, no separate data sharing agreement was required for the present secondary analysis. The present analysis aimed to re-evaluate the dataset using multivariable logistic regression and ML models to identify sociodemographic determinants of knowledge and attitude levels and to explore ML-based classification performances.

### 2.2 Study population

The final dataset included 2,125 students enrolled across the nine academic faculties of Sultan Qaboos University. Participants represented a broad range of academic disciplines and degree levels. Available sociodemographic variables included sex, age group, marital status, academic degree, faculty affiliation, and foundation program enrollment status. These variables were used as independent predictors in the present secondary analysis.

### 2.3 Data collection and instrument structure

The original questionnaire consisted of five sections. The first section provided study information, including the purpose, procedures, voluntary nature of participation, and ethical approval details. The second section obtained informed consent from participants before proceeding to the survey items. The third section collected sociodemographic data, including sex, age, residence, faculty affiliation, marital status, source of information about organ transplantation, and year of study. The fourth section assessed knowledge regarding organ donation, including awareness of organ donation, age limits, benefits and risks, donation of whole or partial organs, existence of national programs, and organ registry systems. The fifth section evaluated attitudes toward organ donation, focusing on perceived life-saving benefits, willingness to register as a donor, donation after death, promotion of donation among relatives, and perceived barriers to donation. The questionnaire incorporated both positively and negatively worded items to reduce response bias and minimize the likelihood of patterned or inattentive responses.

### 2.4 Measures

#### 2.4.1 Assessment of knowledge of organ donation

Knowledge regarding organ donation and transplantation was assessed using 15 structured items with three response options: “Yes,” “No,” and “I do not know.” Each correct response was assigned one point, whereas incorrect and “I do not know” responses were scored as zero. Thus, total knowledge scores ranged from 0 to 15, with higher scores indicating greater knowledge. Consistent with the original scoring framework, knowledge levels were dichotomized using a 60% threshold. Participants scoring between 0 and 8 points were classified as having poor knowledge, while those scoring between 9 and 15 points were categorized as having good knowledge (5). This binary categorization facilitated subsequent inferential analyses and ML-based predictive modeling.

#### 2.4.2 Assessment of attitude toward organ donation

Attitude toward organ donation was evaluated using eight structured items. Scoring followed the same principle applied to the knowledge scale: positive attitude responses were assigned one point, whereas negative or uncertain responses were scored as zero. Total attitude scores therefore ranged from 0 to 8. Based on a 60% cutoff, participants scoring 0–4 points were classified as having a low attitude level, and those scoring 5–8 points were categorized as having a high attitude level (5). In addition to the core attitude items, two multiple-choice questions examining motivational factors for supporting organ donation and reasons for refusal were analyzed descriptively; however, these items were not incorporated into the composite attitude score.

For both knowledge and attitude outcomes, the 60% cutoff was retained to ensure consistency with the original scoring framework and comparability with the source study. This threshold should be interpreted as a pragmatic classification cutoff rather than a threshold externally validated against a clinical or behavioral outcome. Similar pragmatic cutoff approaches, including Bloom’s cutoff-based classifications and thresholds around 60–70%, have been used in many knowledge, attitude, and practice (KAP) and other questionnaire-based studies (33–37).

### 2.5 Ethical considerations and funding

This study was conducted in accordance with the ethical principles of the Declaration of Helsinki (1964) and its subsequent amendments, and complied with all applicable institutional and national regulations governing research involving human participants. The original study was approved by the Medical Research Ethics Committee, College of Medicine and Health Sciences, Sultan Qaboos University, Oman, with ethical approval number EC/397/2021, and written informed consent was obtained from all participants by the original investigators. Ethical approval for the present secondary analysis was also obtained from the Inonu University Institutional Review Board for Non-Interventional Studies (Approval Date: 23 January 2024; Approval Number: 5384). No financial support was received for the design, conduct, data collection, secondary analysis, statistical analysis, interpretation of results, or preparation of this manuscript. Publication-related institutional support, including article processing charge support, was provided by the Inonu University Scientific Research Projects Coordination Unit (Project ID: TSA-2026-4763). The study was designed, analyzed, and reported in accordance with the STROBE (Strengthening the Reporting of Observational Studies in Epidemiology) guidelines to enhance transparency, reproducibility, and methodological rigor (38).

### 2.6 Statistical analysis

All statistical analyses were performed using IBM SPSS Statistics for Windows, Version 25.0 (IBM Corp., Armonk, NY, USA). Categorical variables were summarized as frequencies and percentages. Associations between sociodemographic characteristics and knowledge and attitude levels were evaluated using the Pearson chi-square test. When expected cell counts were small, Yates’s corrected chi-square test was applied. A two-sided *p*-value of <0.05 was considered statistically significant. All statistical tests were two-tailed. Knowledge level and attitude level were treated as dependent categorical variables. Multivariable binary logistic regression analyses were performed to identify independent predictors of good knowledge and high attitude levels. Variables with *p* < 0.05 in the chi-square analyses were considered for inclusion in the multivariable logistic regression models using the enter method. Adjusted odds ratios (aORs) with 95% confidence intervals (CIs) were calculated. Model fit was assessed using the Hosmer–Lemeshow goodness-of-fit test, and explanatory power was evaluated using Cox & Snell and Nagelkerke *R*^2^ statistics.

In addition to conventional regression analysis, supervised ML models were implemented to predict knowledge level and attitude level using variables derived from the study dataset. ML modeling was performed using Python programming language (Python Software Foundation, Wilmington, DE, USA) with the scikit-learn and XGBoost ML libraries for model development and evaluation. Predictor variables were conceptually grouped into three categories: demographic characteristics including age group, sex, and academic degree; motivation factors representing reasons supporting organ donation such as altruistic motivation, perceived life-saving benefit, media influence, religious alignment, and other supportive motivations; and refusal factors representing reasons for declining organ donation such as fear related to organ donation, distrust in medical management, perceived religious prohibition, lack of awareness, and other refusal motivations. Knowledge level and attitude level were defined as binary outcome variables. To evaluate predictive generalizability, the dataset was partitioned using stratified sampling into training (80%) and testing (20%) subsets to assess model performance on an independent validation dataset. All ML models were trained using the same training dataset, and their predictive performance was evaluated on the independent test dataset. Three ensemble ML models were implemented: Random Forest, Extreme Gradient Boosting (XGBoost), and AdaBoost. In the Random Forest model, 100 decision trees were constructed (n_estimators = 100) with random_state = 42 to ensure reproducibility, and node splitting was based on the Gini impurity criterion. In the XGBoost model, 100 estimators (n_estimators = 100) were implemented with the evaluation metric defined as mlogloss, where the gradient boosting framework sequentially builds decision trees to minimize prediction error. In the AdaBoost model, 100 weak learners (n_estimators = 100) were trained using decision tree classifiers as base estimators, and the boosting mechanism iteratively emphasized previously misclassified observations.

Model performance was evaluated using multiple classification metrics including accuracy, sensitivity, specificity, precision, recall, F1-score, and ROC-AUC, each reported with 95% confidence intervals. Accuracy was defined as the proportion of correctly classified observations among all cases in the test dataset. These metrics were compared across models to identify the model demonstrating the highest overall predictive performance. Model performance was additionally visualized using confusion matrices, a performance metrics comparison chart, and ROC curves. To enhance interpretability of the ML models, model-based feature-importance analysis was additionally conducted. This analysis quantified the relative contribution of each predictor variable and ranked them according to importance scores, enabling identification of the most influential demographic characteristics, motivation factors, and refusal factors associated with knowledge and attitude prediction.

## 3 Results

### 3.1 Sociodemographic characteristics of the study population

Table 1 shows that the study population consisted predominantly of female students (68.1%), while males represented 31.9% of participants. The majority of respondents were aged ≤24 years (93.1%), and most were single (94.9%). Nearly all participants were not enrolled in the foundation program (97.1%). In terms of academic degree, 94.5% were pursuing a bachelor’s degree or MD, whereas master’s and doctoral students accounted for 4.6 and 0.8%, respectively. Regarding faculty affiliation, the largest proportions were from the Faculty of Science (17.9%), Arts and Social Sciences (14.2%), and Education (13.7%).

**Table 1:** Sociodemographic characteristics of the study population.

Variables	Categories	Number (%)
Sex	Male	678 (31.9)
	Female	1,447 (68.1)
Age Group (years)	≤24	1978 (93.1)
	>24	147 (6.9)
Marital status	Single	2017 (94.9)
	Married	104 (4.9)
	Divorced	4 (0.2)
Foundation Program Enrollment	Yes	62 (2.9)
	No	2063 (97.1)
Academic degree	Bachelor’s Degree or MD	2009 (94.5)
	Master’s Degree	98 (4.6)
	Doctoral Degree	18 (0.8)
Faculty	Medicine and Health Sci	225 (10.6)
	Engineering	222 (10.4)
	Agricultural and Marine Sci	149 (7.0)
	Economics and Political Sci	270 (12.7)
	Nursing	127 (6.0)
	Law	159 (7.5)
	Science	380 (17.9)
	Education	291 (13.7)
	Arts and Social Sci	302 (14.2)

### 3.2 Knowledge, awareness, and sources of information on organ donation and brain death

Table 2 demonstrates that overall awareness of organ donation was very high, with 98.5% of students reporting that they had heard about organ donation and 95.3% acknowledging that organ donation saves lives. However, only 22.2% knew someone who had donated an organ, and 37.6% knew someone who had received or was awaiting transplantation. Knowledge of specific medical aspects varied: 65.6% correctly identified the kidney as the most frequently transplanted organ, and 82.1% were aware that post-transplant rejection is possible. In contrast, only 14.6% correctly understood ABO blood group compatibility requirements. Religious and legal awareness appeared limited, as 51.0% recognized that Islamic rulings permit organ donation and 47.9% were aware of national legislation. Although 70.8% had heard of brain death, only 12.6% knew that brain death is irreversible. Online sources and social networks were the primary information sources (84.1%), followed by family and friends (27.3%) and radio and television (25.0%).

**Table 2:** Knowledge and awareness regarding organ donation and brain death.

Variable	Category	Number (%)
Awareness of organ donation	Yes	2093 (98.5)
	No	32 (1.5)
	I do not know	0 (0.0)
Belief that organ donation saves lives	Yes	2026 (95.3)
	No	21 (1.0)
	I do not know	78 (3.7)
Knowing someone who has donated an organ	Yes	472 (22.2)
	No	1,653 (77.8)
	I do not know	0 (0.0)
Knowing someone who has received or is awaiting organ transplantation	Yes	800 (37.6)
	No	1,325 (62.4)
	I do not know	0 (0.0)
Knowledge that the kidney is the most frequently transplanted organ worldwide	Yes	1,395 (65.6)
	No	30 (1.4)
	I do not know	700 (32.9)
Awareness that post-transplant organ rejection is possible	Yes	1744 (82.1)
	No	41 (1.9)
	I do not know	340 (16.0)
Knowledge of ABO blood group compatibility requirements in organ transplantation	Yes	311 (14.6)
	No	933 (43.9)
	I do not know	881 (41.5)
Awareness that Islamic religious rulings permit organ donation	Yes	1,083 (51.0)
	No	70 (3.3)
	I do not know	972 (45.7)
Awareness of national legislation regarding organ donation and transplantation	Yes	1,017 (47.9)
	No	44 (2.1)
	I do not know	1,064 (50.1)
Belief that organ donation is ethically acceptable	Yes	1,568 (73.8)
	No	99 (4.7)
	I do not know	458 (21.6)
Awareness of brain death	Yes	1,504 (70.8)
	No	621 (29.2)
	I do not know	0 (0.0)
Awareness of organ donation from brain-dead individuals	Yes	323 (15.2)
	No	498 (23.4)
	I do not know	1,304 (61.4)
Awareness of organ donor cards for brain-dead individuals	Yes	324 (15.2)
	No	1801 (84.8)
	I do not know	0 (0.0)
Knowledge that brain death involves cessation of brainstem reflexes	Yes	426 (20.0)
	No	516 (24.3)
	I do not know	1,183 (55.7)
Knowledge that brain death is irreversible	Yes	268 (12.6)
	No	567 (26.7)
	I do not know	1,290 (60.7)
College-based information sources	Yes	330 (15.5)
	No	1795 (84.5)
Health care facilities	Yes	452 (21.3)
	No	1,673 (78.7)
Internet, online sources, and social networks	Yes	1788 (84.1)
	No	337 (15.9)
Newspapers	Yes	203 (9.6)
	No	1922 (90.4)
Posters	Yes	261 (12.3)
	No	1864 (87.7)
Organ donation campaigns	Yes	464 (21.8)
	No	1,661 (78.2)
Family and friends	Yes	580 (27.3)
	No	1,545 (72.7)
Radio and television	Yes	532 (25.0)
	No	1,593 (75.0)
Other sources	Yes	57 (2.7)
	No	2068 (97.3)

### 3.3 Attitudes toward organ donation

Table 3 indicates that attitudes toward organ donation were generally favorable in principle but inconsistent in practice. A total of 75.0% expressed willingness to donate organs after death, and 68.0% would donate a kidney to a family member with renal failure. Similarly, 68.7% reported willingness to accept a kidney from a deceased donor if needed. However, acceptance of donation from non-relatives was markedly lower (16.2%). Only 28.5% intended to register as organ donors after death, and 34.8% were willing to encourage family members to register. Notably, uncertainty was particularly high regarding organ donation involving financial benefit (68.6%). Similarly, more than half of the students (52.7%) were unsure about their potential future need for transplantation.

**Table 3:** Attitudes toward organ donation.

Variable	Category	Number (%)
Willingness to donate organs after death	Yes	1,594 (75.0)
	No	88 (4.1)
	I do not know	443 (20.8)
Willingness to donate a kidney to a family member with renal failure	Yes	1,445 (68.0)
	No	190 (8.9)
	I do not know	490 (23.1)
Willingness to accept a kidney from a deceased donor if needed	Yes	1,459 (68.7)
	No	211 (9.9)
	I do not know	455 (21.4)
Acceptance of organ donation from non-relatives	Yes	344 (16.2)
	No	1,337 (62.9)
	I do not know	444 (20.9)
Acceptance of organ donation involving financial benefit	Yes	310 (14.6)
	No	357 (16.8)
	I do not know	1,458 (68.6)
Perceived likelihood of needing an organ transplant in the future	Yes	606 (28.5)
	No	400 (18.8)
	I do not know	1,119 (52.7)
Intention to register as an organ donor after death	Yes	606 (28.5)
	No	400 (18.8)
	I do not know	1,119 (52.7)
Willingness to encourage family members to register as organ donors	Yes	740 (34.8)
	No	414 (19.5)
	I do not know	971 (45.7)

### 3.4 Factors motivating support for organ donation

Table 4 summarizes that the primary motivations for supporting organ donation were altruistic and religiously aligned considerations. The perception that organ donation saves lives and aligns with religious beliefs was reported by 76.8% of participants, while 43.9% indicated altruistic motivation as a reason for support. In contrast, personal influence from knowing a donor (8.3%) or someone requiring transplantation (7.0%) was relatively limited. Only 3.9% reported financial motivation as a reason for donation, suggesting that economic incentives were not a dominant driver of positive attitudes.

**Table 4:** Factors motivating support for organ donation.

Variable	Category	Number (%)
Altruistic motivation (desire to help others)	Yes	932 (43.9)
	No	1,193 (56.1)
Perception that organ donation saves lives and aligns with religious beliefs	Yes	1,633 (76.8)
	No	492 (23.2)
Personal influence of knowing an organ donor (family member or friend)	Yes	176 (8.3)
	No	1949 (91.7)
Personal influence of knowing someone who required transplantation	Yes	148 (7.0)
	No	1977 (93.0)
Willingness to donate only to a close relative or loved one	Yes	597 (28.1)
	No	1,528 (71.9)
Positive influence of mass media on the decision to donate	Yes	460 (21.6)
	No	1,665 (78.4)
Belief that Islamic teachings permit organ donation	Yes	661 (31.1)
	No	1,464 (68.9)
Financial motivation	Yes	82 (3.9)
	No	2043 (96.1)
Other reasons	Yes	101 (4.8)
	No	2024 (95.2)

### 3.5 Reasons for refusal of organ donation

Table 5 illustrates that lack of awareness (58.9%) and fear (42.6%) were the most commonly reported reasons for refusal of organ donation. Additionally, 30.0% expressed distrust in physicians or medical management, and 13.2% were concerned that donated organs might not be used appropriately. Religious prohibition was cited by only 5.4% of participants, indicating that religious objection was less prominent than informational and trust-related barriers.

**Table 5:** Reasons for refusal of organ donation.

Variable	Category	Number (%)
Fear related to organ donation	Yes	905 (42.6)
	No	1,220 (57.4)
Lack of trust in physicians or medical management	Yes	637 (30.0)
	No	1,488 (70.0)
Concern that donated organs may not be used appropriately	Yes	280 (13.2)
	No	1845 (86.8)
Perceived irrelevance or lack of personal concern	Yes	118 (5.6)
	No	2007 (94.4)
Perceived religious prohibition (Islamic beliefs)	Yes	114 (5.4)
	No	2011 (94.6)
Lack of awareness regarding organ donation	Yes	1,251 (58.9)
	No	874 (41.1)
Other reasons	Yes	181 (8.5)
	No	1944 (91.5)

### 3.6 Factors associated with knowledge level

Table 6 shows that, overall, 374 students (17.6%) were classified as having good knowledge, whereas 1,751 students (82.4%) were categorized as having poor knowledge. Knowledge levels were significantly associated with academic degree (*p* = 0.042) and faculty affiliation (*p* = 0.033). Students enrolled in bachelor’s degree or MD programs demonstrated higher proportions of good knowledge than postgraduate students. Faculty-based differences were observed, with students from Nursing and Law showing relatively higher proportions of good knowledge compared to several other faculties. In contrast, no statistically significant associations were found between knowledge level and sex (*p* = 0.515), age group (*p* = 0.098), marital status (*p* = 0.999), or foundation program enrollment (*p* = 0.591).

**Table 6:** Association between sociodemographic variables and knowledge level regarding organ donation.

Variables	Categories	Knowledge levels		***p***
		Good (*n* = 374)	Poor (*n* = 1751)	
Sex	Male	114 (30.5)	564 (32.2)	0.515^*^
	Female	260 (69.5)	1,187 (67.8)	
Age group (years)	≤24	356 (95.2)	1,622 (92.6)	0.098^**^
	>24	18 (4.8)	129 (7.4)	
Marital status	Single	363 (97.1)	1,654 (94.5)	0.999^*^
	Married	11 (2.9)	93 (5.3)	
	Divorced	0 (0.0)	4 (0.2)	
Foundation program enrollment	Yes	13 (3.5)	49 (2.8)	0.591**
	No	361 (96.5)	1702 (97.2)	
Academic degree	Bachelor’s Degree or MD	363 (97.1)	1,646 (94.0)	0.042^*^
	Master’s Degree	8 (2.1)	90 (5.1)	
	Doctoral Degree	3 (0.8)	15 (0.9)	
Faculty	Medicine and Health Sci	44 (11.8)	181 (10.3)	0.033^*^
	Engineering	37 (9.9)	185 (10.6)	
	Agricultural and Marine Sci	29 (7.8)	120 (6.9)	
	Economics and Political Sci	45 (12.0)	225 (12.8)	
	Nursing	32 (8.6)	95 (5.4)	
	Law	40 (10.7)	119 (6.8)	
	Science	57 (15.2)	323 (18.4)	
	Education	44 (11.8)	247 (14.1)	
	Arts and Social Sci	46 (12.3)	256 (14.6)	

Table 7 summarizes the distribution of supporting motivations according to knowledge level of study group. Students with good knowledge more frequently reported knowing an organ donor (11.8% vs. 7.5%, *p* = 0.007), knowing someone who required transplantation (10.7% vs. 6.2%, *p* = 0.002), and believing that Islamic teachings permit organ donation (36.6% vs. 29.9%, *p* = 0.011). No significant differences were observed for altruistic motivation (*p* = 0.109), perception that donation saves lives (*p* = 0.317), willingness to donate only to relatives (*p* = 0.258), media influence (*p* = 0.165), financial motivation (*p* = 0.643), or other reasons (*p* = 0.634).

**Table 7:** Supporting motivations for organ donation according to knowledge level.

Variables	Categories	Knowledge levels		***p***
		Good (*n* = 374)	Poor (*n* = 1751)	
Altruistic motivation (desire to help others)	Yes	178 (47.6)	754 (43.1)	0.109
	No	196 (52.4)	997 (56.9)	
Perception that organ donation saves lives and aligns with religious beliefs	Yes	280 (74.9)	1,353 (77.3)	0.317
	No	94 (25.1)	398 (22.7)	
Personal influence of knowing an organ donor (family member or friend)	Yes	44 (11.8)	132 (7.5)	0.007
	No	330 (88.2)	1,619 (92.5)	
Personal influence of knowing someone who required transplantation	Yes	40 (10.7)	108 (6.2)	0.002
	No	334 (89.3)	1,643 (93.8)	
Willingness to donate only to a close relative or loved one	Yes	114 (30.5)	483 (27.6)	0.258
	No	260 (69.5)	1,268 (72.4)	
Positive influence of mass media on the decision to donate	Yes	91 (24.3)	369 (21.1)	0.165
	No	283 (75.7)	1,382 (78.9)	
Belief that Islamic teachings permit organ donation	Yes	137 (36.6)	524 (29.9)	0.011
	No	237 (63.4)	1,227 (70.1)	
Financial motivation	Yes	16 (4.3)	66 (3.8)	0.643
	No	358 (95.7)	1,685 (96.2)	
Other reasons	Yes	16 (4.3)	85 (4.9)	0.634
	No	358 (95.7)	1,666 (95.1)	

Table 8 compares refusal reasons by knowledge level. Most refusal factors did not differ significantly between groups. Among the refusal-related factors, only “other reasons” showed a statistically significant association with knowledge level (11.5% vs. 7.9%, *p* = 0.023). Fear (*p* = 0.587), distrust in physicians (*p* = 0.543), concern about misuse (*p* = 0.194), perceived irrelevance (*p* = 0.660), religious prohibition (*p* = 0.813), and lack of awareness (*p* = 0.079) were not significantly associated with knowledge level.

**Table 8:** Refusal reasons for organ donation according to knowledge level.

Variables	Categories	Knowledge levels		*p*
		Good (*n* = 374)	Poor (*n* = 1751)	
Fear related to organ donation	Yes	164 (43.9)	741 (42.3)	0.587
	No	210 (56.1)	1,010 (57.7)	
Lack of trust in physicians or medical management	Yes	117 (31.3)	520 (29.7)	0.543
	No	257 (68.7)	1,231 (70.3)	
Concern that donated organs may not be used appropriately	Yes	57 (15.2)	223 (12.7)	0.194
	No	317 (84.8)	1,528 (87.3)	
Perceived irrelevance or lack of personal concern	Yes	19 (5.1)	99 (5.7)	0.660
	No	355 (94.9)	1,652 (94.3)	
Perceived religious prohibition (Islamic beliefs)	Yes	21 (5.6)	93 (5.3)	0.813
	No	353 (94.4)	1,658 (94.7)	
Lack of awareness regarding organ donation	Yes	205 (54.8)	1,046 (59.7)	0.079
	No	169 (45.2)	705 (40.3)	
Other reasons	Yes	43 (11.5)	138 (7.9)	0.023
	No	331 (88.5)	1,613 (92.1)	

### 3.7 Multivariable logistic regression analysis for knowledge level

Table 9 indicated that a binary logistic regression analysis was performed to identify independent predictors of good knowledge regarding organ donation. The overall model was statistically significant (Omnibus χ^2^ = 29.10, *p* < 0.001), and the Hosmer–Lemeshow test demonstrated good model fit (χ^2^ = 1.24, *p* = 0.872). However, the explained variance was low (Cox and Snell *R*^2^ = 0.014; Nagelkerke *R*^2^ = 0.022), indicating limited predictive strength. In the multivariable model, academic degree was significantly associated with knowledge level overall (*p* = 0.041). Compared with participants holding a Bachelor’s Degree/MD, those with a Master’s degree had significantly lower odds of good knowledge (OR = 0.39, *p* = 0.012), whereas having a PhD degree was not significantly associated with knowledge level (OR = 0.88, *p* = 0.845). Knowing someone who required transplantation was independently associated with higher odds of good knowledge (OR = 1.64, *p* = 0.020). Belief that Islamic teachings permit organ donation was independently associated with higher odds of good knowledge (OR = 1.29, *p* = 0.033). Reporting other reasons was also positively associated with good knowledge (OR = 1.53, *p* = 0.023). In contrast, knowing an organ donor (family member or friend) was not statistically significant after adjustment (OR = 1.33, *p* = 0.154). Although the overall classification accuracy was 82.4%, the model showed limited ability to correctly identify individuals with good knowledge, indicating limited discriminative performance despite acceptable overall fit.

**Table 9:** Multivariable logistic regression analysis identifying independent predictors of knowledge level.

Variables	***B***	SE	Wald	*p*	Exp *B*	95%CI
Academic Degree (overall)	—	—	6.38	0.041	—	—
Academic Degree (Master’s vs. Bachelor/MD)	−0.95	0.38	6.35	0.012	0.39	0.19–0.81
Academic Degree (PhD vs. Bachelor/MD)	−0.13	0.64	0.04	0.845	0.88	0.25–3.08
Personal influence of knowing an organ donor (family member or friend)	0.29	0.20	2.03	0.154	1.33	0.90–1.97
Personal influence of knowing someone who required transplantation	0.49	0.21	5.41	0.020	1.64	1.08–2.48
Belief that Islamic teachings permit organ donation	0.26	0.12	4.55	0.033	1.29	1.02–1.64
Other reasons for refusing organ donation	0.43	0.19	5.20	0.023	1.53	1.06–2.21
Constant	−1.33	0.27	24.16	<0.001	0.26	—

### 3.8 Factors associated with attitude level

Table 10 shows that, overall, 608 students (28.6%) were classified as having a high attitude level, whereas 1,517 students (71.4%) were categorized as having a low attitude level. Attitude levels were not significantly associated with any of the examined sociodemographic variables. No statistically significant relationships were observed between attitude level and sex (*p* = 0.257), age group (*p* = 0.859), marital status (*p* = 0.872), foundation program enrollment (*p* = 0.724), academic degree (*p* = 0.882), or faculty affiliation (*p* = 0.409), indicating that favorable or unfavorable attitudes were relatively uniformly distributed across demographic categories.

**Table 10:** Association between sociodemographic variables and attitude level toward organ donation.

Variables	Categories	Attitude levels		*p*
		High (n = 608)	Low (n = 1,517)	
Sex	Male	205 (33.7)	473 (31.2)	0.257*
	Female	403 (66.3)	1,044 (68.8)	
Age Group (years)	≤ 24	565 (92.9)	1,413 (93.1)	0.859*
	> 24	43 (7.1)	104 (6.9)	
Marital status	Single	575 (94.6)	1,442 (95.1)	0.872*
	Married	32 (5.3)	72 (4.7)	
	Divorced	1 (0.2)	3 (0.2)	
Foundation Program Enrollment	Yes	16 (2.6)	46 (3.0)	0.724**
	No	592 (97.4)	1,471 (97.0)	
Academic degree	Bachelor’s Degree or MD	573 (94.2)	1,436 (94.7)	0.882*
	Master’s Degree	29 (4.8)	69 (4.5)	
	Doctoral Degree	6 (1.0)	12 (0.8)	
Faculty	Medicine and Health Sci	62 (10.2)	163 (10.7)	0.409*
	Engineering	66 (10.9)	156 (10.3)	
	Agricultural and Marine Sci	55 (9.0)	94 (6.2)	
	Economics and Political Sci	83 (13.7)	187 (12.3)	
	Nursing	32 (5.3)	95 (6.3)	
	Law	41 (6.7)	118 (7.8)	
	Science	100 (16.4)	280 (18.5)	
	Education	84 (13.8)	207 (13.6)	
	Arts and Social Sci	85 (14.0)	217 (14.3)	

*: Pearson chi square test; **: Yates’s corrected chi-square test.

Table 11 compares supporting motivations according to attitude level. Students with high attitude levels were significantly more likely to report altruistic motivation (52.1% vs. 40.5%, *p* < 0.001), the perception that donation saves lives and aligns with religious beliefs (89.1% vs. 71.9%, *p* < 0.001), positive media influence (32.9% vs. 17.1%, *p* < 0.001), knowing someone who required transplantation (8.7% vs. 6.3%, *p* = 0.045), and belief that Islamic teachings permit donation (43.8% vs. 26.0%, *p* < 0.001). Conversely, willingness to donate only to close relatives was more common among students with low attitude levels (32.2% vs. 17.9%, *p* < 0.001). Financial motivation showed a modest association (p = 0.045), whereas knowing an organ donor (*p* = 0.813) and other reasons (*p* = 0.982) were not statistically significant.

**Table 11:** Supporting motivations for organ donation according to attitude level.

Variables	Categories	Attitude levels		***p***
		High (*n* = 608)	Low (*n* = 1,517)	
Altruistic motivation (desire to help others)	Yes	317 (52.1)	615 (40.5)	<0.001
	No	291 (47.9)	902 (59.5)	
Perception that organ donation saves lives and aligns with religious beliefs	Yes	542 (89.1)	1,091 (71.9)	<0.001
	No	66 (10.9)	426 (28.1)	
Personal influence of knowing an organ donor (family member or friend)	Yes	49 (8.1)	127 (8.4)	0.813
	No	559 (91.9)	1,390 (91.6)	
Personal influence of knowing someone who required transplantation	Yes	53 (8.7)	95 (6.3)	0.045
	No	555 (91.3)	1,422 (93.7)	
Willingness to donate only to a close relative or loved one	Yes	109 (17.9)	488 (32.2)	<0.001
	No	499 (82.1)	1,029 (67.8)	
Positive influence of mass media on the decision to donate	Yes	200 (32.9)	260 (17.1)	<0.001
	No	408 (67.1)	1,257 (82.9)	
Belief that Islamic teachings permit organ donation	Yes	266 (43.8)	395 (26.0)	<0.001
	No	342 (56.3)	1,122 (74.0)	
Financial motivation	Yes	32 (5.3)	50 (3.3)	0.045
	No	576 (94.7)	1,467 (96.7)	
Other reasons	Yes	29 (4.8)	72 (4.7)	0.982
	No	579 (95.2)	1,445 (95.3)	

Table 12 presents refusal reasons according to attitude level. Students with low attitude levels more frequently reported fear (45.1% vs. 36.3%, *p* < 0.001), perceived irrelevance (6.9% vs. 2.1%, *p* < 0.001), religious prohibition (6.3% vs. 3.1%, *p* = 0.004), and lack of awareness (68.2% vs. 35.5%, *p* < 0.001). Distrust in physicians (*p* = 0.199) and concern about misuse of organs (*p* = 0.386) were not significantly associated with attitude level.

**Table 12:** Refusal reasons for organ donation according to attitude level.

Variables	Categories	Attitude levels		***p***
		High (*n* = 608)	Low (*n* = 1,517)	
Fear related to organ donation	Yes	221 (36.3)	684 (45.1)	<0.001
	No	387 (63.7)	833 (54.9)	
Lack of trust in physicians or medical management	Yes	170 (28.0)	467 (30.8)	0.199
	No	438 (72.0)	1,050 (69.2)	
Concern that donated organs may not be used appropriately	Yes	74 (12.2)	206 (13.6)	0.386
	No	534 (87.8)	1,311 (86.4)	
Perceived irrelevance or lack of personal concern	Yes	13 (2.1)	105 (6.9)	<0.001
	No	595 (97.9)	1,412 (93.1)	
Perceived religious prohibition (Islamic beliefs)	Yes	19 (3.1)	95 (6.3)	0.004
	No	589 (96.9)	1,422 (93.7)	
Lack of awareness regarding organ donation	Yes	216 (35.5)	1,035 (68.2)	<0.001
	No	392 (64.5)	482 (31.8)	
Other reasons	Yes	115 (18.9)	66 (4.4)	<0.001
	No	493 (81.1)	1,451 (95.6)	

### 3.9 Multivariable logistic regression analysis for attitude level

Table 13 indicated that a binary logistic regression analysis was performed to determine whether both supporting and refusal-related factors were independently associated with high attitude toward organ donation. The overall model was statistically significant (Omnibus χ^2^ = 467.19, *p* < 0.001). The Hosmer–Lemeshow test was not significant (χ^2^ = 13.06, *p* = 0.110), indicating good model fit. The explained variance was moderate (Cox and Snell *R*^2^ = 0.197; Nagelkerke *R*^2^ = 0.283), demonstrating improved predictive capacity compared with the previous model. In the multivariable analysis, several factors were independently associated with higher odds of high attitude. Altruistic motivation significantly increased the likelihood of high attitude (OR = 1.60, *p* < 0.001). The perception that organ donation saves lives and aligns with religious beliefs emerged as one of the strongest predictors (OR = 2.60, *p* < 0.001). Positive influence of mass media was also significantly associated with higher attitude (OR = 1.91, *p* < 0.001). Belief that Islamic teachings permit organ donation independently increased the odds of high attitude (OR = 1.61, *p* < 0.001). Financial motivation showed a strong positive association as well (OR = 2.70, *p* < 0.001). Additionally, other reasons for refusing organ donation were positively associated with high attitude (OR = 1.66, *p* = 0.011). Conversely, several factors were independently associated with lower odds of high attitude. Lack of awareness regarding organ donation was the strongest negative predictor (OR = 0.23, *p* < 0.001). Perceived irrelevance or lack of personal concern significantly reduced the likelihood of high attitude (OR = 0.28, *p* < 0.001). Perceived religious prohibition (OR = 0.43, *p* = 0.003), fear related to organ donation (OR = 0.55, *p* < 0.001), and willingness to donate only to a close relative (OR = 0.56, *p* < 0.001) were also associated with decreased odds of high attitude. Personal influence of knowing someone who required transplantation was not statistically significant in the adjusted model (*p* = 0.209). The overall classification accuracy was 77.4%, indicating a substantial improvement compared with the constant-only model.

**Table 13:** Multivariable logistic regression analysis identifying independent predictors of attitude level.

Variables	***B***	SE	Wald	*p*	Exp *B*	95%CI
Altruistic motivation (desire to help others)	0.47	0.11	18.28	<0.001	1.60	1.29–1.99
Perception that organ donation saves lives and aligns with religious beliefs	0.95	0.16	36.41	<0.001	2.60	1.90–3.54
Personal influence of knowing someone who required transplantation	0.27	0.21	1.58	0.209	1.31	0.86–1.98
Willingness to donate only to a close relative or loved one	−0.59	0.14	18.23	<0.001	0.56	0.43–0.73
Positive influence of mass media on the decision to donate	0.65	0.13	25.87	<0.001	1.91	1.49–2.45
Belief that Islamic teachings permit organ donation	0.48	0.12	16.84	<0.001	1.61	1.28–2.03
Financial motivation	1.00	0.28	13.03	<0.001	2.70	1.58–4.64
Fear related to organ donation	−0.59	0.12	23.77	<0.001	0.55	0.44–0.70
Perceived irrelevance or lack of personal concern	−1.27	0.32	15.48	<0.001	0.28	0.15–0.53
Perceived religious prohibition (Islamic beliefs)	−0.84	0.29	8.61	0.003	0.43	0.25–0.76
Lack of awareness regarding organ donation	−1.47	0.12	148.59	<0.001	0.23	0.18–0.29
Other reasons for refusing organ donation	0.50	0.20	6.46	0.011	1.66	1.12–2.44
Constant	−1.33	0.26	25.25	<0.001	0.27	-

### 3.10 Ensemble ML models for predicting knowledge level

**Figure 1:**
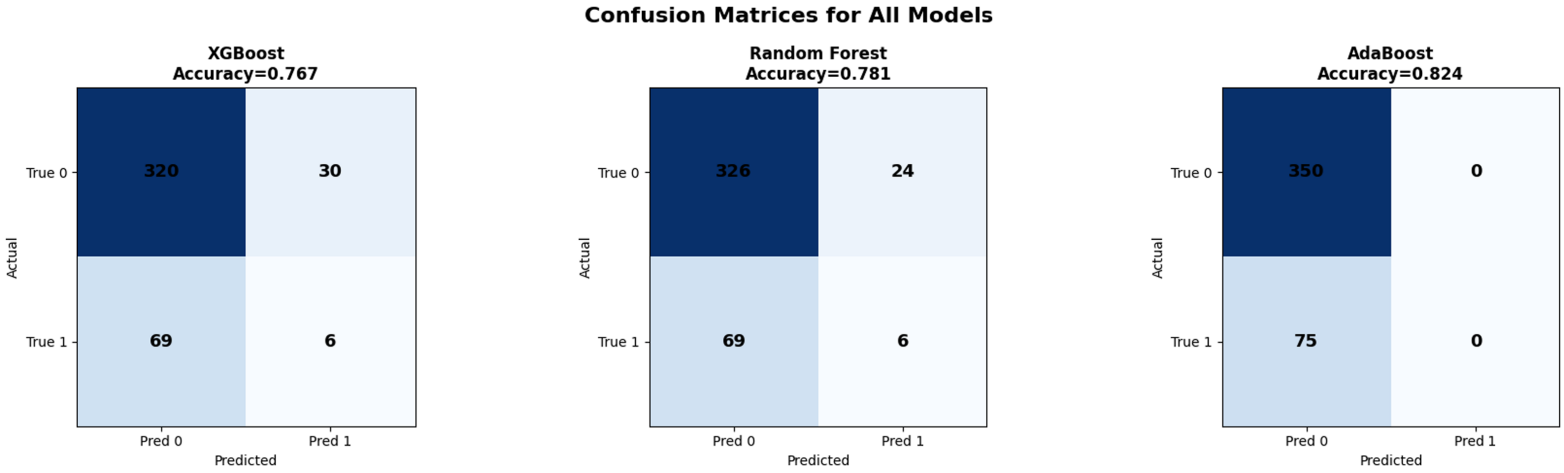
Comparison of confusion matrices for ML models predicting knowledge level.

**Figure 2:**
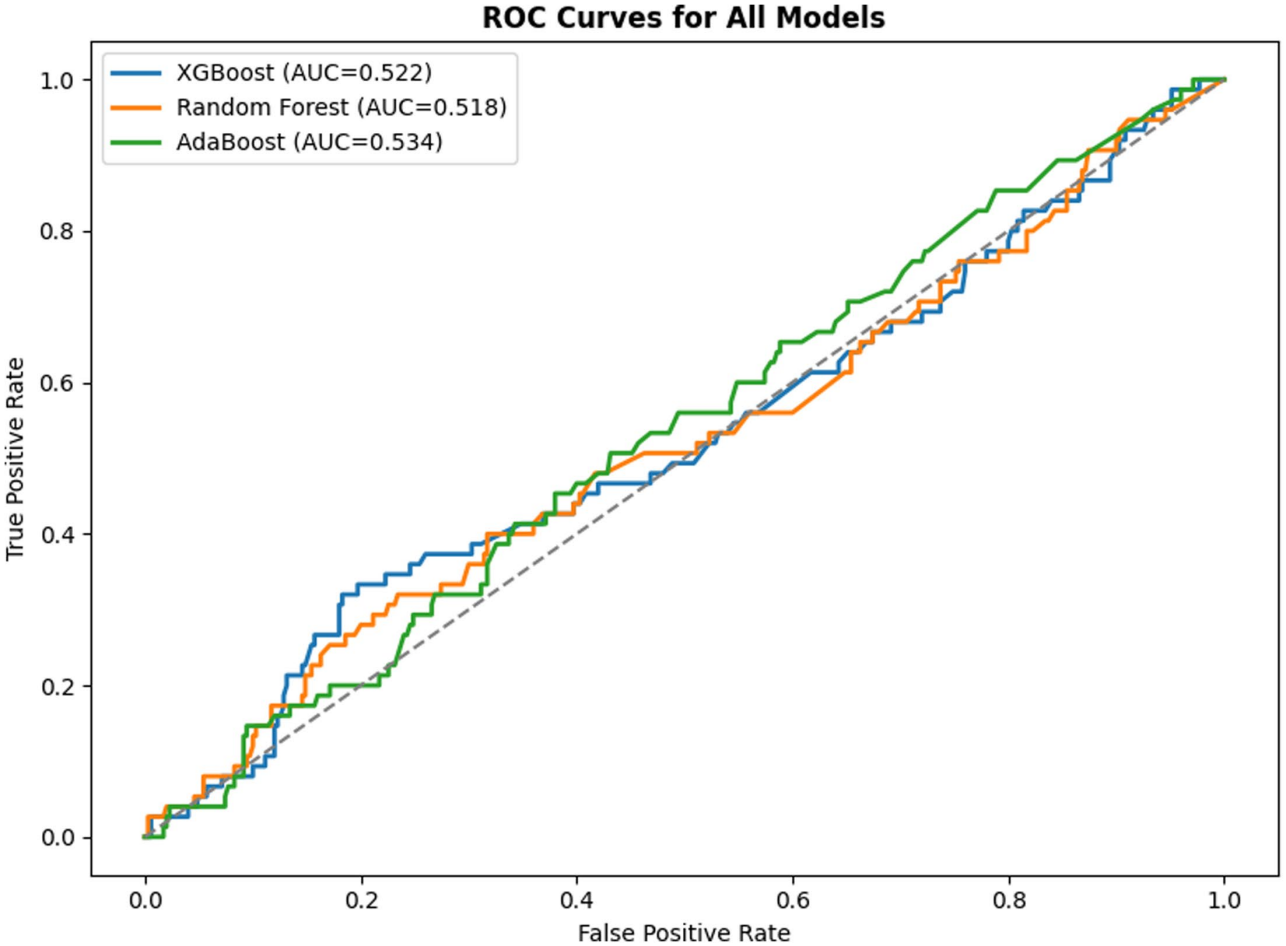
ROC analysis of ML models for knowledge level prediction.

**Figure 3:**
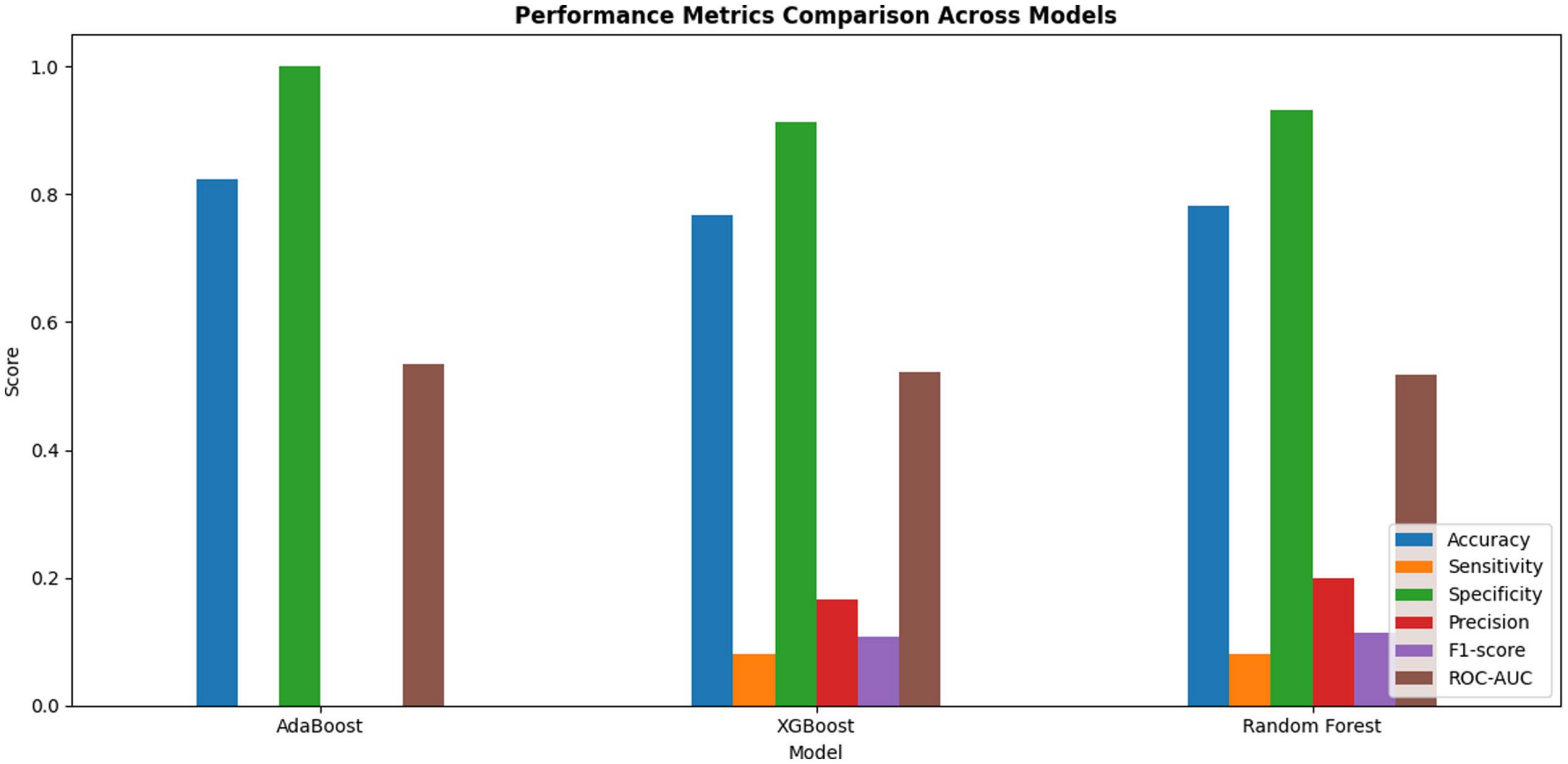
Performance metrics comparison of ML models for knowledge level prediction.

**Figure 4:**
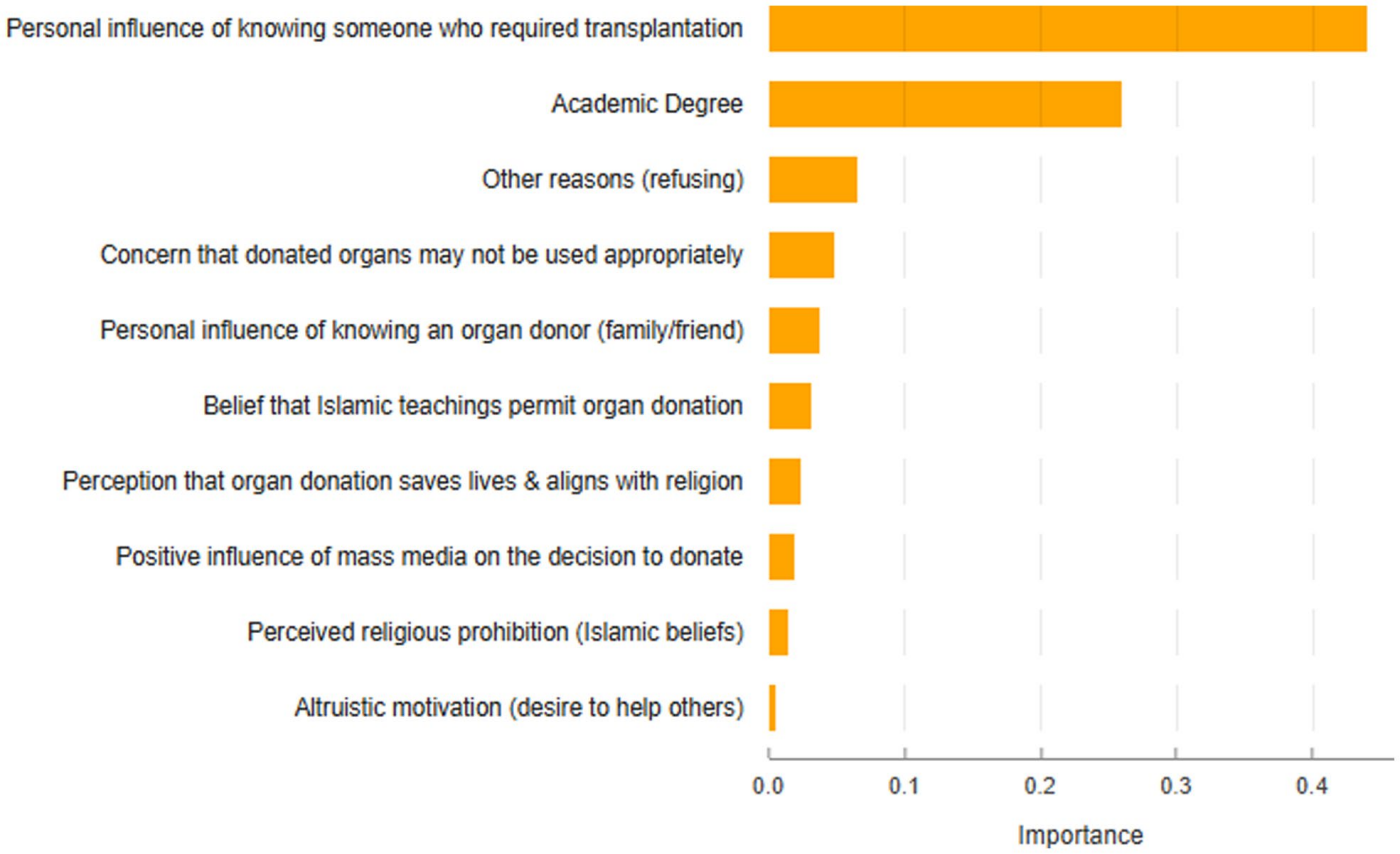
Feature importance of predictor variables in the AdaBoost model for predicting knowledge level.

For knowledge-level prediction, the classification accuracies were 0.82 (95% CI: 0.79–0.86) for AdaBoost, 0.77 (95% CI: 0.73–0.81) for XGBoost, and 0.78 (95% CI: 0.74–0.82) for Random Forest. Detailed performance metrics are presented in Table 14, and the results are shown in Figures 1–3. Model-based feature-importance analysis identified the personal influence of knowing someone who required transplantation as the most influential predictor of knowledge level, with a markedly higher importance score than all remaining variables. Academic degree ranked second, reflecting a meaningful contribution of educational background to the classification process. Among refusal-related variables, other reasons for refusing organ donation and concern that donated organs may not be used appropriately showed relatively higher importance scores. Among supporting motivations, personal influence of knowing an organ donor (family member or friend), belief that Islamic teachings permit organ donation, perception that organ donation saves lives and aligns with religious beliefs, and positive influence of mass media on the decision to donate showed moderate contributions to the prediction task, whereas altruistic motivation and perceived religious prohibition demonstrated comparatively lower influence (Figure 4). Overall, these findings suggest that experiential exposure to transplantation, educational level, and specific refusal- and motivation-related perceptions are the main contributors to knowledge level classification in this dataset.

**Table 14:** ML model performance metrics for predicting knowledge level (with 95% CI).

Model	AdaBoost	XGBoost	Random Forest
Accuracy	0.82 (0.79–0.86)	0.77 (0.73–0.81)	0.78 (0.74–0.82)
Sensitivity	0.00 (0.00–0.00)	0.08 (0.03–0.14)	0.08 (0.03–0.14)
Specificity	1.00 (1.00–1.00)	0.91 (0.88–0.94)	0.93 (0.90–0.96)
Precision	Not estimable	0.17 (0.06–0.30)	0.20 (0.07–0.36)
Recall	0.00 (0.00–0.00)	0.08 (0.03–0.14)	0.08 (0.03–0.14)
F1-score	Not estimable	0.11 (0.04–0.19)	0.11 (0.04–0.20)
ROC-AUC	0.53 (0.47–0.60)	0.52 (0.44–0.59)	0.52 (0.44–0.59)

Precision and F1-score were not estimable for AdaBoost because the model generated no positive-class predictions for knowledge-level classification.

### 3.11 Ensemble ML models for predicting attitude level

**Figure 5:**
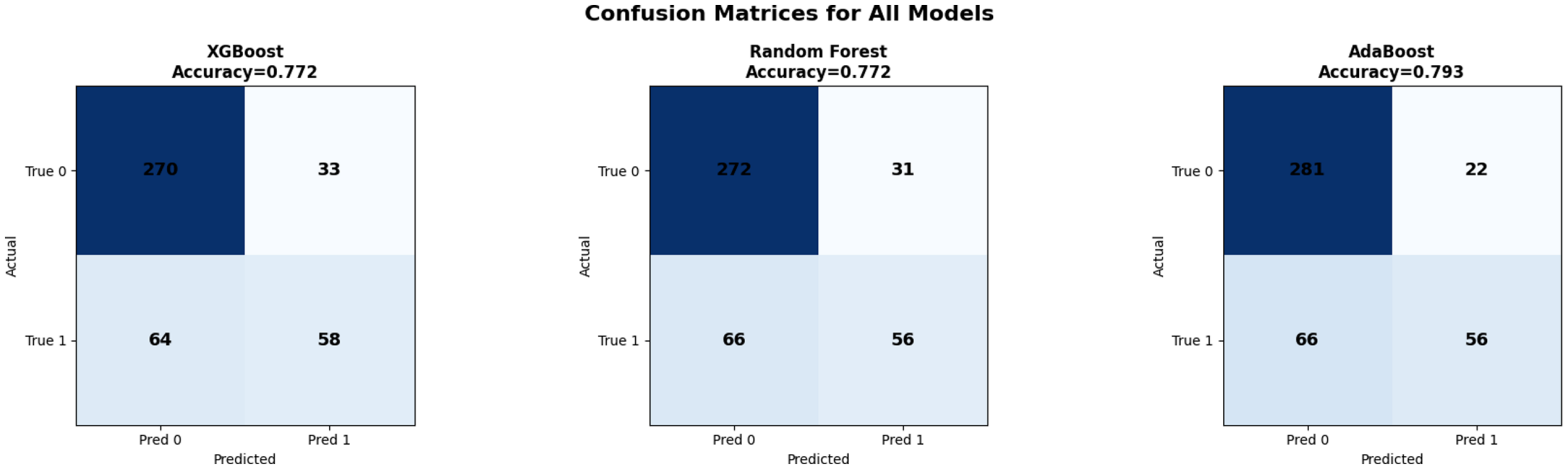
Comparison of confusion matrices for ML models predicting attitude level.

**Figure 6:**
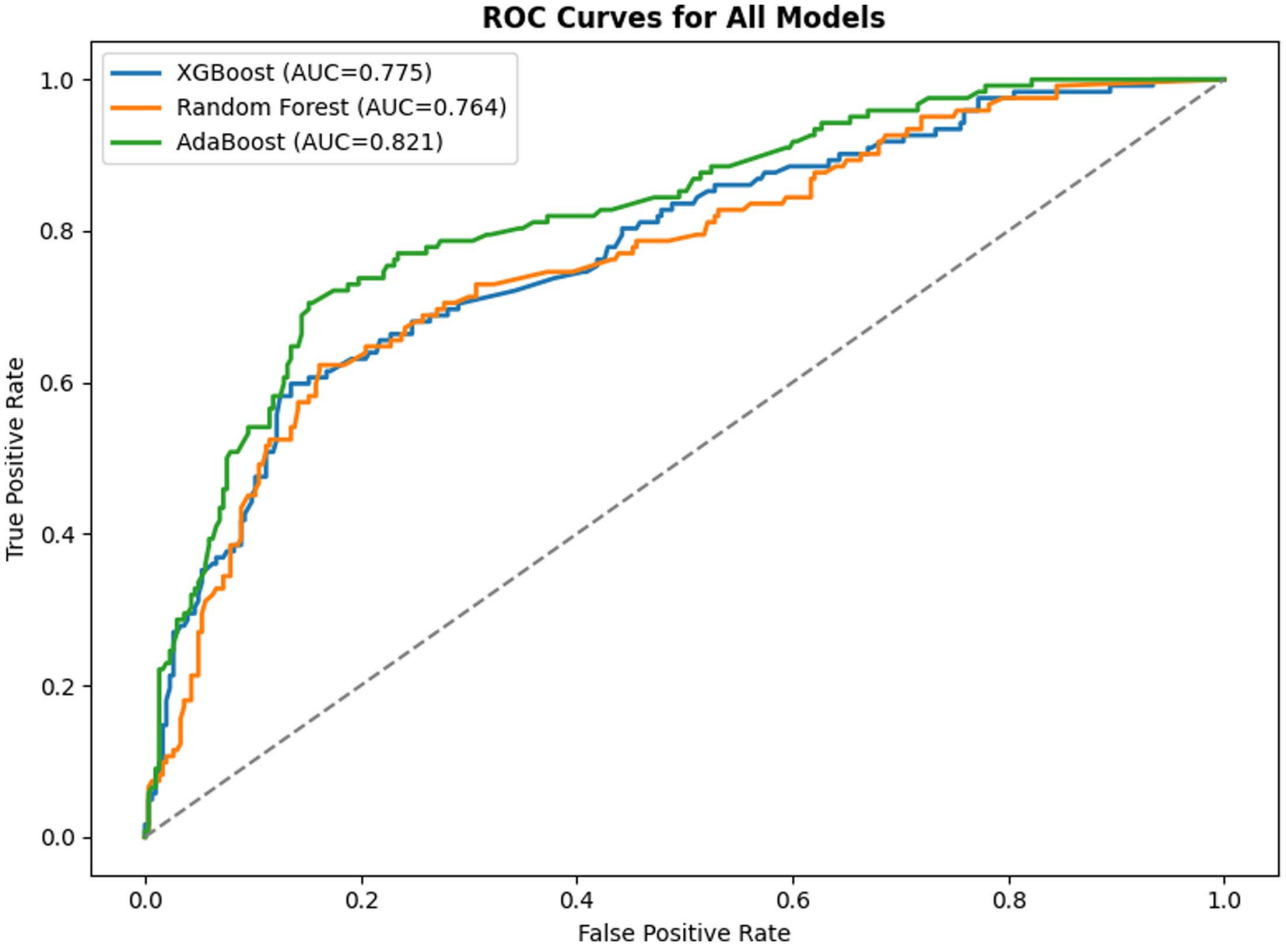
ROC analysis of ML models for attitude level prediction.

**Figure 7:**
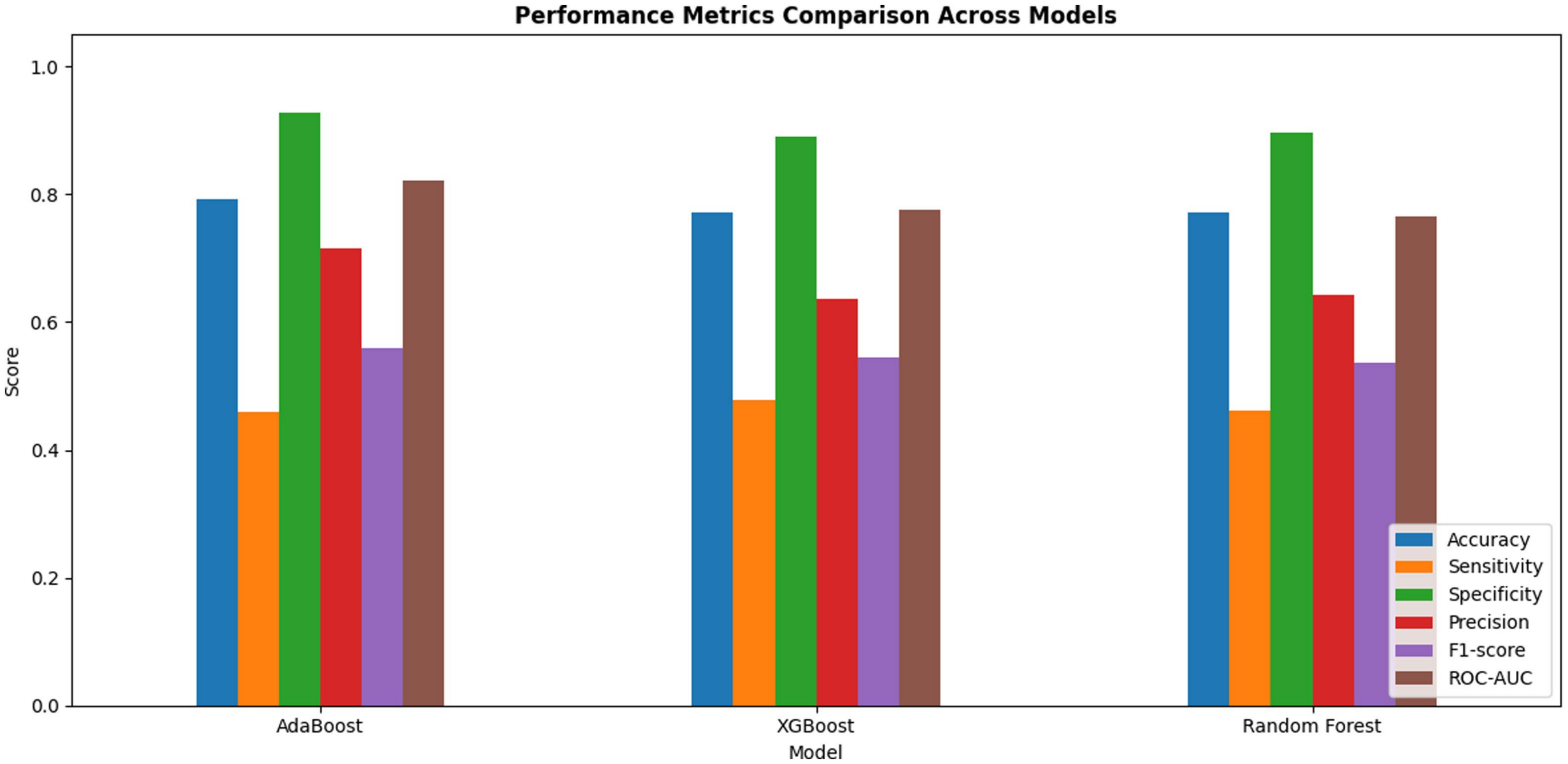
Performance metrics comparison of ML models for attitude level prediction.

**Figure 8:**
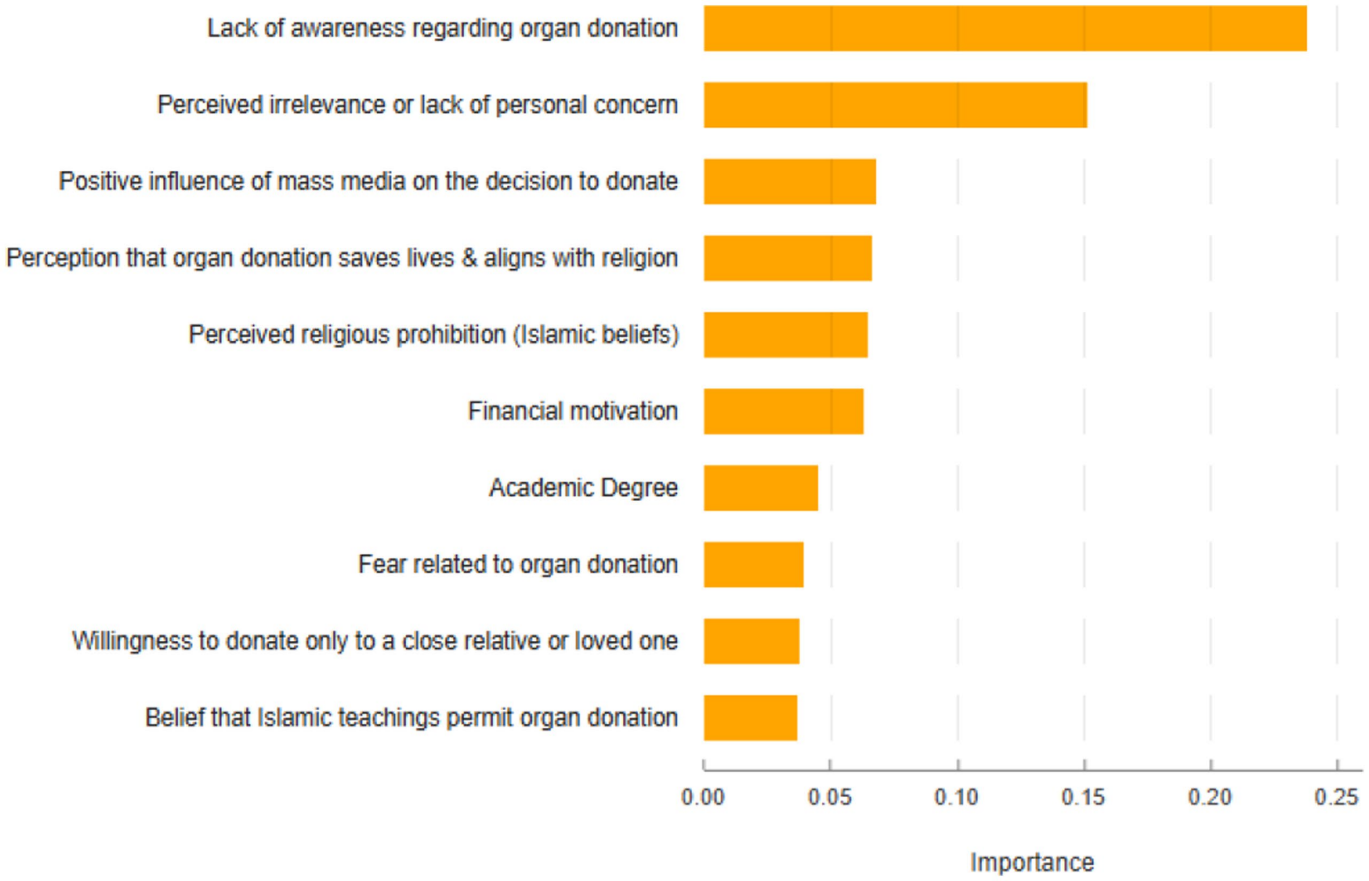
Feature importance of predictor variables in the AdaBoost model for predicting attitude level.

For attitude-level prediction, the classification accuracies were 0.79 (95% CI: 0.76–0.83) for AdaBoost, 0.77 (95% CI: 0.73–0.81) for XGBoost, and 0.77 (95% CI: 0.73–0.81) for Random Forest. Detailed performance metrics are presented in Table 15, and the results are shown in Figures 5–7. Model-based feature-importance analysis indicated that the most influential predictor for attitude level was the lack of awareness regarding organ donation, which exhibited the highest importance score among all predictors included in the model. The second most influential factor was perceived irrelevance or lack of personal concern, highlighting the substantial role of refusal-related perceptions in shaping predicted attitude levels. Among supporting motivation variables, positive influence of mass media on the decision to donate and perception that organ donation saves lives and aligns with religious beliefs showed relatively higher contributions. Perceived religious prohibition and financial motivation also demonstrated notable importance, followed by academic degree, fear related to organ donation, willingness to donate only to a close relative or loved one, and belief that Islamic teachings permit organ donation. Although demographic variables such as academic degree contributed to the prediction process, their relative influence was lower compared with behavioral and perception-based variables (Figure 8). Taken together, these findings indicate that refusal-related perceptions made the largest contribution to attitude-level prediction in this dataset, while supporting motivations and educational characteristics also contribute to the predictive framework, highlighting the importance of both positive motivations and perceived barriers in shaping students’ attitudes toward organ donation.

**Table 15:** ML model performance metrics for predicting attitude level (with 95% CI).

Model	AdaBoost	XGBoost	Random Forest
Accuracy	0.79 (0.76–0.83)	0.77 (0.73–0.81)	0.77 (0.73–0.81)
Sensitivity	0.46 (0.37–0.55)	0.48 (0.39–0.56)	0.46 (0.37–0.55)
Specificity	0.93 (0.90–0.95)	0.89 (0.86–0.93)	0.90 (0.86–0.93)
Precision	0.72 (0.62–0.81)	0.64 (0.54–0.74)	0.64 (0.55–0.75)
Recall	0.46 (0.37–0.55)	0.48 (0.39–0.56)	0.46 (0.37–0.55)
F1-score	0.56 (0.47–0.64)	0.55 (0.46–0.62)	0.54 (0.45–0.62)
ROC-AUC	0.82 (0.78–0.87)	0.78 (0.73–0.83)	0.77 (0.71–0.82)

### 3.12 Structured comparison of key predictors identified by multivariable logistic regression and AdaBoost feature-importance analysis

Table 16 presents a structured comparison of key predictors identified by multivariable logistic regression and AdaBoost feature-importance analysis. For knowledge-level prediction, partial overlap was observed between the two approaches. Knowing someone who required transplantation, academic degree, other reasons for refusing organ donation, and belief that Islamic teachings permit organ donation were identified in the regression model and also contributed to AdaBoost-based classification. In contrast, concern that donated organs may not be used appropriately and knowing an organ donor were not significant independent predictors in the regression model but showed measurable contribution in AdaBoost feature-importance analysis.

**Table 16:** Structured comparison of overlapping and method-specific predictors identified by multivariable logistic regression and AdaBoost feature-importance analysis.

Outcome	Predictor/domain	Logistic regression finding	AdaBoost feature-importance finding	Interpretation
Knowledge	Knowing someone who required transplantation	Positive predictor of good knowledge	Highest importance	Overlapping finding
Knowledge	Academic degree	Predictor of knowledge level	High importance	Partially overlapping finding
Knowledge	Other reasons for refusing organ donation	Positive predictor of good knowledge	Important refusal-related predictor	Overlapping finding; requires cautious interpretation
Knowledge	Belief that Islamic teachings permit organ donation	Positive predictor of good knowledge	Lower importance	Partially overlapping finding
Knowledge	Concern that donated organs may not be used appropriately	Not retained in the final regression model	Important refusal-related predictor	ML-specific finding
Knowledge	Knowing an organ donor	Not significant after adjustment	Modest importance	ML-specific finding
Attitude	Lack of awareness regarding organ donation	Strong negative predictor of high attitude	Highest importance	Overlapping finding
Attitude	Perceived irrelevance or lack of personal concern	Negative predictor of high attitude	High importance	Overlapping finding
Attitude	Positive influence of mass media on the decision to donate	Positive predictor of high attitude	Important predictor	Overlapping finding
Attitude	Perception that organ donation saves lives and aligns with religious beliefs	Positive predictor of high attitude	Important predictor	Overlapping finding
Attitude	Perceived religious prohibition based on Islamic beliefs	Negative predictor of high attitude	Moderate importance	Overlapping finding
Attitude	Financial motivation	Positive predictor of high attitude	Moderate importance	Overlapping finding
Attitude	Academic degree	Not retained in final regression model	Moderate importance	ML-specific finding
Attitude	Fear related to organ donation	Negative predictor of high attitude	Lower importance	Partially overlapping finding
Attitude	Willingness to donate only to a close relative or loved one	Negative predictor of high attitude	Lower importance	Partially overlapping finding
Attitude	Belief that Islamic teachings permit organ donation	Positive predictor of high attitude	Lower importance	Partially overlapping finding
Attitude	Altruistic motivation	Positive predictor of high attitude	Not among leading predictors	Regression-specific finding
Attitude	Other reasons for refusing organ donation	Positive predictor of high attitude	Not among leading predictors	Regression-specific finding; requires cautious interpretation
Attitude	Knowing someone who required transplantation	Not significant after adjustment	Not among leading predictors	No overlapping signal

For attitude-level prediction, overlapping findings were observed for lack of awareness regarding organ donation, perceived irrelevance or lack of personal concern, positive influence of mass media, perception that organ donation saves lives and aligns with religious beliefs, perceived religious prohibition, and financial motivation. Conversely, academic degree contributed to AdaBoost-based classification but was not retained as an independent predictor in the regression model. Altruistic motivation and other reasons for refusing organ donation were identified in the regression model but were not among the leading AdaBoost feature-importance predictors. Overall, the structured comparison indicated partially overlapping findings between the two analytical frameworks, while also showing method-specific predictors. These findings should be interpreted in light of the fact that multivariable logistic regression estimates adjusted associations, whereas AdaBoost feature importance reflects the relative contribution of predictors to classification performance.

## 4 Discussion

University students constitute an important group that reflects general societal tendencies in terms of both educational level and social representation (39). In this context, the present study investigated knowledge and attitudes toward organ donation among university students in Oman, a Muslim-majority country, and explored the individual, religious, and sociocultural factors underlying these outcomes. The results revealed a substantial gap between general awareness of organ donation and structured knowledge and attitudes. To better understand the determinants of this gap, the analysis combined multivariable logistic regression with ensemble ML models. This integrated analytical approach allowed the study to both quantify independent associations between predictors and outcomes and identify broader predictive patterns related to organ donation knowledge and attitudes. These findings should be interpreted within the broader global challenge of organ donation shortages.

Organ donation is one of the most effective treatment methods that prolong survival and improve quality of life for patients with end-stage organ failure. However, a significant imbalance exists worldwide between the supply of and demand for organs. Despite the increasing number of patients on waiting lists, the inability to find a sufficient number of donors results in the death of many patients awaiting transplantation each year. Cultural, ethical, legal, and particularly religious factors are known to play an important role among the causes of organ donation shortage (5). In Muslim societies, attitudes toward organ donation are closely shaped by religious beliefs and cultural values. Although Islam promotes saving human life, individual decisions are often influenced by personal beliefs, values, and social environment (40). Even though religious leaders and institutions support organ donation in many Islamic countries, its practical reflection remains limited due to various reservations.

Within the broader literature on Muslim-majority settings, lower organ donation rates have been linked to religious, cultural, and social factors. However, the findings of the present study should be interpreted specifically within the context of university students in Oman. Some studies have shown that participants hold misconceptions that organ donation is contrary to Islam (5, 41, 42). In addition to these misunderstandings, cultural emphasis on bodily integrity and attitudes against touching the body after death may reinforce reluctance toward organ donation (42). Therefore, examining knowledge and attitudes toward organ donation among university students in Oman provides context-specific insights into the medical, sociocultural, and educational factors shaping organ donation perceptions in this sample.

The vast majority of participants were aware that organ donation is a life-saving act (95.3%), and their knowledge that the kidney is the most frequently transplanted organ was consistent with findings reported in the literature (43). However, when knowledge was evaluated using the structured scoring system applied in this study, only 17.6% of students were classified as having good knowledge, whereas 82.4% were categorized as having poor knowledge. Likewise, only 28.6% demonstrated a high attitude level, while 71.4% were classified as having a low attitude level. This marked discrepancy between general awareness and structured knowledge and attitude scores indicates that superficial familiarity with organ donation does not necessarily translate into comprehensive understanding or positive behavioral orientation. Moreover, only half of the students were aware of religious rulings permitting organ donation within the framework of Islam (21, 44, 45). This finding suggests that lack of religious knowledge may be one of the important factors underlying hesitant or negative attitudes. Similarly, although the proportion of those reporting awareness of brain death was relatively high (70.8%), the proportion of those who knew that organ donation can be performed in cases of brain death was quite low (15.2%). The literature frequently emphasizes that this situation stems from conceptual confusion regarding the ethical, medical, and legal aspects of brain death (46, 47).

Another noteworthy finding of the study concerns sources of information. A total of 84.1% of participants obtained information through digital media and social networks. This proportion indicates that traditional sources such as family (27.3%) and healthcare institutions (21.3%) remain relatively secondary. Previous studies have noted that although media is effective in raising awareness, it has limited influence on actual donation decisions (48–50). In our study, only 21.6% reported considering donation due to media influence. This finding suggests that effective information strategies should rely not only on media campaigns but also on multilayered interventions engaging personal values, social environment, and religious authorities.

A notable feature of the present study is the parallel evaluation of determinants using both multivariable logistic regression and ensemble ML models. This approach enabled a structured comparison of predictors identified by the two analytical frameworks, as summarized in Table 16, and provided a broader interpretation of the factors associated with organ-donation knowledge and attitudes among university students. Multivariable logistic regression estimates adjusted associations and effect sizes, whereas ML models identify variables that contribute to predictive classification, including potential non-linear patterns or interactions. Therefore, the findings from these two approaches should be interpreted as complementary rather than directly equivalent.

Regarding knowledge about organ donation, the multivariable logistic regression analysis identified the independent determinants of good knowledge. Knowing someone who required transplantation significantly increased the likelihood of having a good knowledge level (OR = 1.64), and believing that Islamic teachings permit organ donation was also associated with higher knowledge (OR = 1.29). Reporting “other reasons” for refusing organ donation was positively associated with knowledge level (OR = 1.53), whereas enrollment in a master’s degree program was associated with lower odds of good knowledge (OR = 0.39). Ensemble ML modeling showed partially overlapping findings. The AdaBoost model identified knowing someone who required transplantation, academic degree, concerns regarding the misuse of donated organs, and the perception that organ donation saves lives as the most influential predictors of knowledge classification. The AdaBoost model achieved an accuracy of 0.82 in classifying knowledge level.

Regarding attitudes toward organ donation, the multivariable logistic regression analysis identified the determinants of supportive attitudes. Perceiving organ donation as life-saving and compatible with religious beliefs was the strongest positive determinant (OR = 2.60), followed by altruistic motivation (OR = 1.60) and the positive influence of mass media (OR = 1.91). Belief that Islamic teachings permit organ donation was also positively associated with supportive attitudes (OR = 1.61), while financial motivation showed a strong positive association with favorable attitudes (OR = 2.70). Reporting other reasons for refusing organ donation was likewise associated with higher odds of supportive attitudes (OR = 1.66). Conversely, several factors were associated with lower odds of favorable attitudes, including lack of awareness (OR = 0.23), perceived irrelevance (OR = 0.28), fear related to organ donation (OR = 0.55), willingness to donate only to close relatives (OR = 0.56), and perceived religious prohibition (OR = 0.43). However, knowing someone who required transplantation did not remain statistically significant in the adjusted model. The AdaBoost model achieved the highest accuracy among the evaluated models (0.79) and identified lack of awareness and perceived irrelevance as the most influential predictors, followed by media influence, perception of organ donation as life-saving and religiously acceptable, financial motivation, willingness to donate only to close relatives, and belief in Islamic permissibility.

The positive association observed for “other reasons for refusing organ donation” should be interpreted cautiously. This variable represents a heterogeneous residual category rather than a single predefined refusal barrier. Therefore, it may include additional, nuanced, or non-standard explanations that are not necessarily equivalent to opposition to organ donation. A similar pattern was also observed in the knowledge-level analysis, where “other reasons” was more frequent among participants with good knowledge and remained positively associated with good knowledge in the multivariable model. This consistency across both knowledge and attitude analyses suggests that participants with higher knowledge or more favorable attitudes may have been more likely to report additional explanations beyond the predefined response options. Therefore, future questionnaire-based studies should either avoid using broad residual categories such as “other reasons” or expand the predefined response options and include structured open-ended fields to better capture participants’ individual perspectives and autonomous reasoning.

Previous studies investigating factors influencing organ-donation attitudes and decisions have predominantly relied on conventional statistical approaches such as logistic regression, regression modeling, and correlation analyses. In contrast, studies employing ML models in this field remain relatively limited. For example, Boadu et al. (51) in the United Kingdom applied a Random Forest–based ML model to analyze national survey data and to estimate public intentions to become living kidney donors. Their model identified several important predictors, including support for organ donation, awareness of donation campaigns, gender, age, occupation, religion, household composition, and ethnic origin. The model demonstrated a predictive performance of approximately 71.1% in estimating the propensity for living kidney donation. Khan (52) in the USA applied ML models to predict family consent for organ donation and reported that the XGBoost model achieved the best predictive performance (accuracy = 81.7%). Freda et al. (30) in Italy applied ML models to forecast the likelihood of obtaining consent for organ donation and reported that the AdaBoost model achieved the best predictive performance, with an accuracy of approximately 85%. Khan and Tutun (29) in the USA developed a network-based ML predictive framework to model organ-donation consent outcomes using data from an organ procurement organization. Their results showed that the network-enhanced logistic regression model achieved the best predictive performance, reaching an accuracy of approximately 99.9%, substantially outperforming the conventional ML models tested in the study. These studies collectively indicate that ML models may provide complementary predictive insights into organ-donation decision processes, although their application in this research area remains relatively scarce. In the present study, several ensemble ML models were comparatively evaluated alongside multivariable logistic regression to analyze determinants of organ-donation knowledge and attitudes. Among the evaluated models, the AdaBoost model showed the highest accuracy in our dataset, suggesting its potential utility for classifying organ-donation knowledge and attitude levels.

The ML findings should be interpreted in the context of the imbalanced outcome distributions. This issue was particularly evident for knowledge-level prediction, where the high overall accuracy may partly reflect the predominance of the poor-knowledge class rather than strong discrimination of participants with good knowledge. Therefore, sensitivity, specificity, precision, recall, F1-score, ROC-AUC, and confusion matrix findings were considered together to provide a more balanced interpretation of model behavior. During model development, resampling-based approaches, including SMOTE, were also explored using the training dataset only. However, SMOTE did not provide a consistent improvement in test-set performance and worsened some performance metrics. This finding is consistent with methodological studies showing that class-imbalance correction and SMOTE-based resampling are not universally beneficial; such approaches may fail to improve discrimination, worsen calibration, or generate synthetic observations that do not fully preserve the original minority-class distribution. Therefore, the final interpretation was based on models evaluated under the original outcome distribution and reported using multiple performance metrics (53–57).

Taken together, the comparison of multivariable logistic regression results with findings from ensemble ML models suggests partially overlapping and complementary patterns between the two approaches. Variables such as lack of awareness, religious permissibility, media influence, and experiential exposure were observed across both analytical frameworks, suggesting relevant determinants of organ-donation knowledge and attitudes. At the same time, ML models identified additional contextual predictors that may operate through complex interactions. Collectively, these findings suggest that combining multivariable logistic regression with ensemble ML models provides a more comprehensive understanding of the behavioral and perceptual factors shaping organ-donation attitudes.

### 4.1 Limitations

Several limitations should be considered when interpreting the findings of this study. First, the cross-sectional design prevents causal interpretations of the relationships observed between knowledge, attitudes, and the identified predictors. Second, the study population consisted exclusively of students from a single university in Oman. Therefore, the findings may not be generalizable to students from other Omani universities, the broader Omani population, or other Muslim-majority countries with different sociocultural, educational, religious, and healthcare contexts. Third, knowledge and attitude data were obtained through self-reported questionnaires and may therefore be subject to recall bias or social desirability bias. In addition, the knowledge classification applied in this study was based on a predefined scoring framework, which may not fully capture the multidimensional nature of knowledge related to organ donation. Moreover, the dichotomization of knowledge and attitude scores may have resulted in information loss and reduced variability in the outcome measures. Although the 60% threshold was retained to ensure consistency with the original study and comparability with the source dataset, it was not externally validated against a clinical or behavioral criterion. Alternative thresholds may yield different classifications. However, median-based dichotomization would not eliminate the inherent information loss; rather, it would only shift the cutoff and create a sample-dependent classification. Finally, class imbalance was an important limitation of the ML analyses, particularly for knowledge-level prediction, because only 17.6% of participants were classified as having good knowledge. Although resampling-based approaches, including SMOTE, were explored during model development, they did not provide a consistent improvement in test-set performance and worsened some metrics. Therefore, the final models were interpreted under the original outcome distribution. This approach preserves the natural distribution of the outcome but requires cautious interpretation, because high overall accuracy may reflect majority-class prediction rather than adequate discrimination of the minority class. Future studies using larger datasets, more balanced samples, cost-sensitive learning, or threshold-optimization strategies may provide more reliable minority-class prediction.

## 5 Conclusion

In this Omani university-student sample, awareness of organ donation among students was very high, and most participants recognized that organ donation saves lives; however, structured knowledge and favorable donation attitudes remained limited, and a major gap was observed in understanding brain death. Multivariable logistic regression identified academic degree, knowing someone who required transplantation, belief that Islamic teachings permit organ donation, and other reasons for refusing donation as independent predictors of good knowledge. For attitude level, significant predictors included altruistic motivation, perception that organ donation saves lives and aligns with religion, positive influence of mass media, belief that Islamic teachings permit organ donation, financial motivation, and several perception-related barriers such as fear, perceived irrelevance, perceived religious prohibition, and lack of awareness. These partially overlapping findings suggest that AdaBoost-based feature-importance patterns were broadly aligned with several regression-based determinants. Specifically, the AdaBoost model highlighted personal exposure to transplantation and educational factors as key predictors of knowledge, while lack of awareness and perceived irrelevance were the most influential predictors of attitude.

## Data Availability

The dataset analyzed in this study is publicly available in the supplementary materials of the original open-access publication. Further inquiries can be directed to the corresponding author.
